# Invasive lobular carcinoma with extracellular mucin production and HER-2 overexpression: a case report and further case studies

**DOI:** 10.1186/1746-1596-5-36

**Published:** 2010-06-15

**Authors:** Jing Yu, Rohit Bhargava, David J Dabbs

**Affiliations:** 1 Department of Pathology, Magee-Womens Hospital of University of Pittsburgh Medical Center, Pittsburgh, PA, USA

## Abstract

Invasive lobular carcinomas (ILC) of breast typically demonstrate intracytoplasmic mucin. We present a unique case of classical type ILC with abundant extracellular mucin and strong *ERBB2 *(HER2/neu) expression confirmed by immunohistochemistry and fluorescent in situ hybridization. Dual E-cadherin/p120 immunohistochemical stain demonstrated complete loss of membranous E-cadherin and the presence of diffuse cytoplasmic p120 staining, confirming the lobular phenotype. The tumor cells showed ductal-like cytoplasmic MUC1 staining, but were negative for MUC2 and other mucin gene markers. In addition, studies of tissue microarrays of 80 breast carcinomas with mucinous differentiation revealed 4 pure mucinous carcinomas showing significantly reduced E-cadherin staining without redistribution of p120 into cytoplasm. The findings suggest that the presence of extracellular mucin does not exclude a diagnosis of lobular carcinoma, and the morphologic and molecular characteristics of lobular and ductal carcinomas are more complex than previously appreciated.

## Introduction

Ductal carcinoma and lobular carcinoma are traditionally considered two distinct types of mammary carcinoma with characteristic morphology, immunohistochemical profile, and clinical behavior. The classification of ductal versus lobular carcinoma is routinely based on the growth pattern and cytology of the tumor cells. E-cadherin immunohistochemical stain is used in cases with equivocal morphology.

Invasive lobular carcinomas (ILCs) are characterized by cytologically uniform cells with round nuclei and inconspicuous nucleoli, as well as discohesive architecture with linear or non-linear growth pattern. A variable portion of ILC cells show intracytoplasmic mucin secretion and demonstrate signet-ring cell morphology. Historically, extracellular mucin production is by default a feature of ductal carcinoma. Lobular carcinoma has been considered a variant of mucin-secreting carcinoma with only intracytoplasmic mucin [1-4]. In common practice, a diagnosis of mucinous carcinoma or ductal carcinoma with mucinous features is often made in the presence of extracellular mucin, without immunohistochemical confirmation of the ductal phenotype.

The majority of classical ILCs express estrogen receptor and progesterone receptor, but lack *ERBB2 *(*HER2*) expression or amplification, and therefore, fall into the "luminal" molecular class [5-12]. Rare cases of ILCs with positive HER2 may be seen, and most represent the pleomorphic variant.

We report a unique case of classical ILC with abundant extracellular mucin secretion and unusual expression of mucin (MUC) gene product as well as HER-2 overexpression. In order to investigate whether lobular phenotype is indeed a rare variant of extracellular mucin-producing carcinoma of breast, we also studied tissue microarrays of 80 breast carcinomas with mucinous differentiation, including 40 pure mucinous carcinomas and 40 carcinomas with mixed mucinous and non-mucinous components.

### Clinical History

A 65-year-old postmenopausal woman, who had no family history of breast cancer, presented with a 3 cm spiculated mass in the left breast on a recent mammogram. Ultrasound-guided core biopsy performed in an outside hospital confirmed an invasive ductal carcinoma with abundant mucin production. She was referred to our institution and subsequently underwent a resection of the tumor along with sentinel lymph node biopsy.

## Materials and methods

Tissue specimens obtained from the lumpectomy were fixed a minimum of 8 hours in 10% neutral phosphate-buffered formalin, embedded in paraffin, and sectioned at four microns. The paraffin-embedded sections were stained with hematoxylin-eosin (H&E) for light microscopic examination. Special stains for Mucicarmine and Alcian-Blue were used to confirm the mucin production and its localization.

### Immunohistochemistry

Immunohistochemical studies were performed on 4-μm-thick sections of paraffin-embedded tissue using the Ventana Benchmark XT system (Tuscon, AZ) with iView DAB (2'-diaminobenzamide) Detection Kit. The antibodies with their vendors, clones, and dilutions are listed in Table 1. For dual E-cadherin/p120 stain, cell membrane immunostaining of E-cadherin (detected with UltraView Universal DAB) and cell membrane p120 (detected with UltraView Universal RED) staining were considered to be ductal phenotype, whereas complete loss of the membranous E-cadherin staining and the presence of diffuse cytoplasmic p120 staining without membranous accentuation were considered to be lobular phenotype, as described in our previous study [13]. Estrogen receptor (ER) and progesterone receptor (PR) were considered positive if more than 1% of the tumor cells revealed nuclear staining. HER2/neu was scored on a 0 to 3+ scale using the College of American Pathologist (CAP)/American Society of Clinical Oncology (ASCO) criteria. Internal and external positive and negative controls accompanied the hormone receptors and Her2 analyses.

**Table 1:** Antibodies used for immunohistochemistry

Antibody	Clone	Dilution	Source
E-cadherin	ECH-6	1:100	Ventana (Tucson, AZ)
p120	98	1:200	BD Biosciences (San Diego, CA)
ER	SP1	Prediluted	Ventana (Tucson, AZ)
PR	1E2	Prediluted	Ventana (Tucson, AZ)
c-erbB-2	CB11	Prediluted	Ventana (Tucson, AZ)
Ki-67	30-9	Prediluted	Ventana (Tucson, AZ)
MUC1	Ma695	1:100	Vector (Burlingame, CA)
MUC2	Ccp58	1:25	Vector (Burlingame, CA)
MUC4	1G8	1:100	Zymed (San Francisco, CA)
MUC5AC	CLH2	1:100	Vector (Burlingame, CA)
MUC6	CLH5	1:25	Vector (Burlingame, CA)

### Fluorescence in situ hybridization

Fluorescence in situ hybridization (FISH) analysis of *HER2*gene was performed on a formalin-fixed block using PathVysion dual color *HER-2 *DNA Probe Kit (Vysis, Downers Grove, IL). At least 30 non-overlapping interphase tumor cell nuclei were evaluated. In each nucleus, the number of *HER2*signals and chromosome 17 centromere signals (D17Z1) were counted. The HER2/CEP17 ratio was calculated. A ratio of greater than 2.2 is considered to be amplified according to recent ASCO-CAP guidelines.

### Tissue Microarray

Tissue microarrays (TMAs) with threefold redundancy were created for 40 cases of pure mucinous carcinoma and 40 cases of carcinomas with mixed mucinous and non-mucinous components. Three to six tissue cores each with a core diameter of 0.6 mm punched from representative tumor regions of each donor block were transferred and arrayed into a new recipient paraffin blocks using a tissue microarrayer (Beecher Instruments, Sun Prairie, WI, USA).

## Results

### Histologic findings

**Figure 1:**
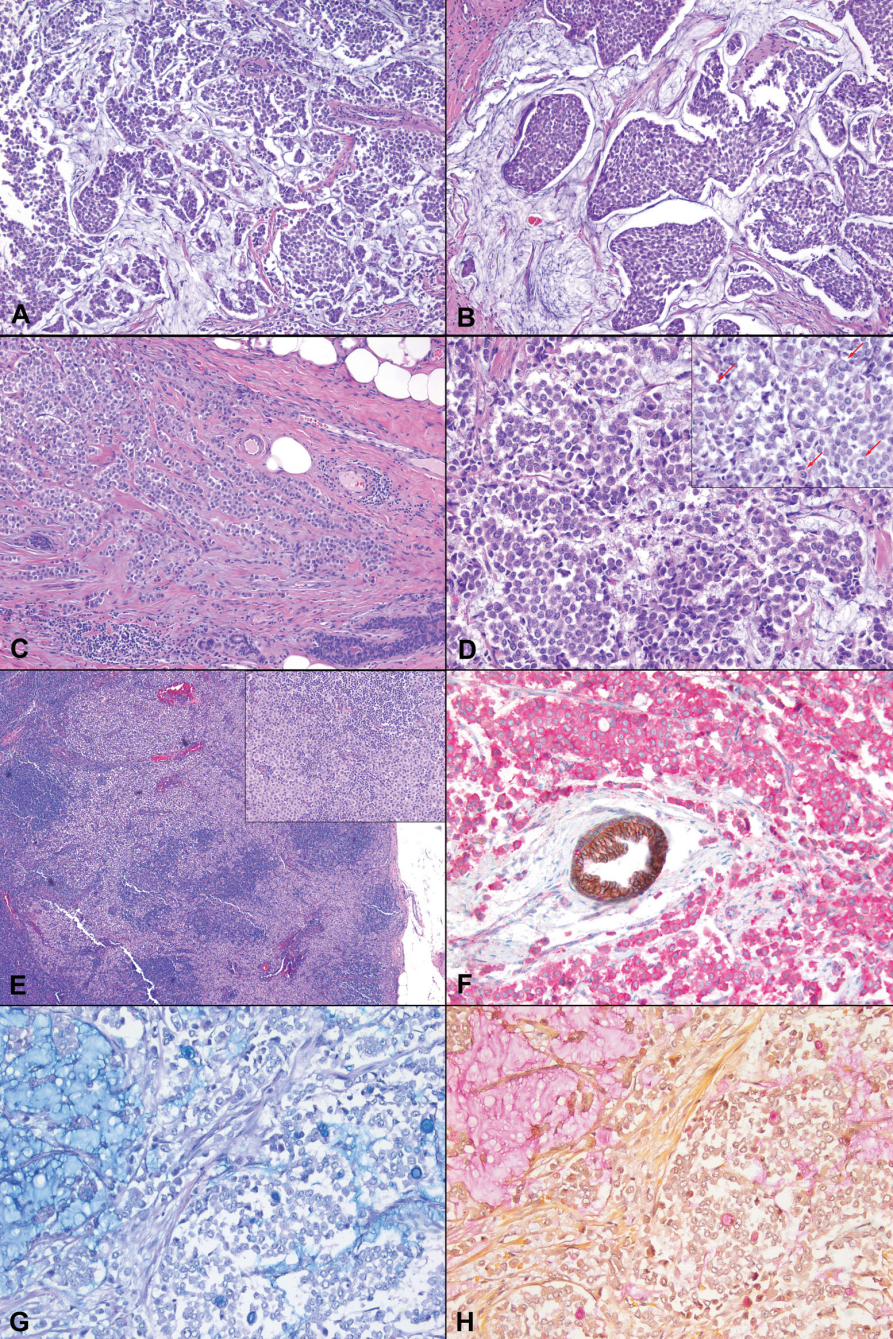
**A case of invasive lobular carcinoma of breast with extracellular mucin**. The tumor showed discohesive (A, H&E, ×100) and nested (B, ×200) growth with abundant extracellular mucin. At the periphery, the tumor cells formed cords and single files (C, ×100). Cytologically, the tumor cells were small to medium in size and relatively uniform (D, ×400), with scattered signet ring cells present (D inset, arrows, ×400). A sentinel lymph node showed metastatic focus of uniform tumor cells (E inset, ×40) in dissociated infiltrating pattern (E, ×200). Immunohistochemical stain revealed complete absence of membranous E-cadherin staining (brown) and the presence of diffuse cytoplasmic p120 staining (pink); a normal duct with membranous staining for both E-cadherin and p120 served as an internal control (F, immunohistochemistry, ×400). Alcian Blue (G, ×200) and Mucicarmine (H, ×200) stains highlighted both extracellular and intracellular mucin.

Microscopic examination revealed a tumor with predominantly solid and nested growth pattern, which intervened with abundant extracellular mucinous material (Figure 1). In the peripheral areas where minimal mucin was present, the tumor formed infiltrating cords and single files. The tumor cells were small to medium in size, relatively uniform and round, with small nucleoli and scant to moderate amount of cytoplasm. Signet-ring cells with intracytoplasmic vacuoles were readily seen. Foci of classical lobular carcinoma in situ were present away from the invasive component. One of the sentinel lymph nodes demonstrated a 3.5 mm focus of metastasis with uniform tumor cells in dissociated infiltrating pattern without associated mucin, characteristic of metastatic lobular carcinoma in a lymph node (Figure 1). Special stains for Mucicarmine and Alcian Blue highlighted extracellular as well as intracellular mucin (Figure 1).

### Immunohistochemical findings

**Figure 2:**
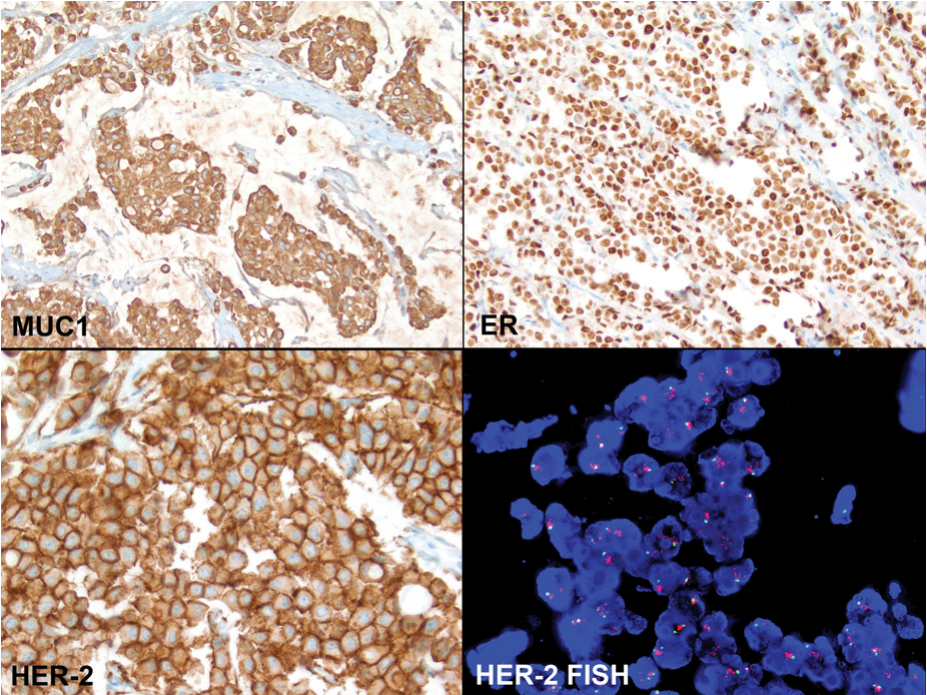
**Characteristics of the invasive lobular carcinoma with extracellular mucin**. The tumor cells revealed strong cytoplasmic MUC1 staining (top left, immunohistochemistry, ×200), but were negative for MUC2, MUC4, MUC5AC and MUC6 (images not shown). The tumor cells were positive for ER (top right, ×400), negative for PR (image not shown), and positive for HER-2/neu with 3+ staining (bottom left, ×400). FISH study confirmed the amplification of *HER-2 *gene (bottom right, ×1,000).

Dual E-cadherin/p120 stain was performed to characterize the phenotype of the tumor (Figure 2). The internal control of the normal duct showed membranous staining of both E-cadherin (brown) and p120 (red). The tumor cells demonstrated complete absence of membranous E-cadherin staining but diffuse cytoplasmic p120 staining, unequivocally confirming the lobular phenotype.

The lobular carcinoma cells were diffusely and strongly positive for ER, but negative for PR. Uncommonly seen in lobular carcinomas, the tumor cells revealed an unambiguous diffuse 3+ HER-2 staining (Figure 2). Ki-67 stain showed approximately 25% proliferation index.

To evaluate the expression profile of mucin in this unusual case, a panel of immunohistochemical stains for mucin (MUC) gene products was performed. Essentially all the tumor cells demonstrated strong cytoplasmic MUC1 staining (Figure 2), but were negative for MUC2, MUC4, MUC5AC and MUC6.

### FISH findings

The ratio of HER-2/neu signals to chromosome 17 centromere signals was >8.74. Since a ratio of greater than 2.2 is considered amplified, this specimen was unequivocally amplified for *HER2 *gene (Figure 2). The average number of *HER-2 *signals per cell was >12.68. The average number of signals for the chromosome 17 centromere was 1.45. Overall, the FISH findings were typical of a HER2 immunohistochemical 3+ case.

### TMA findings

**Figure 3:**
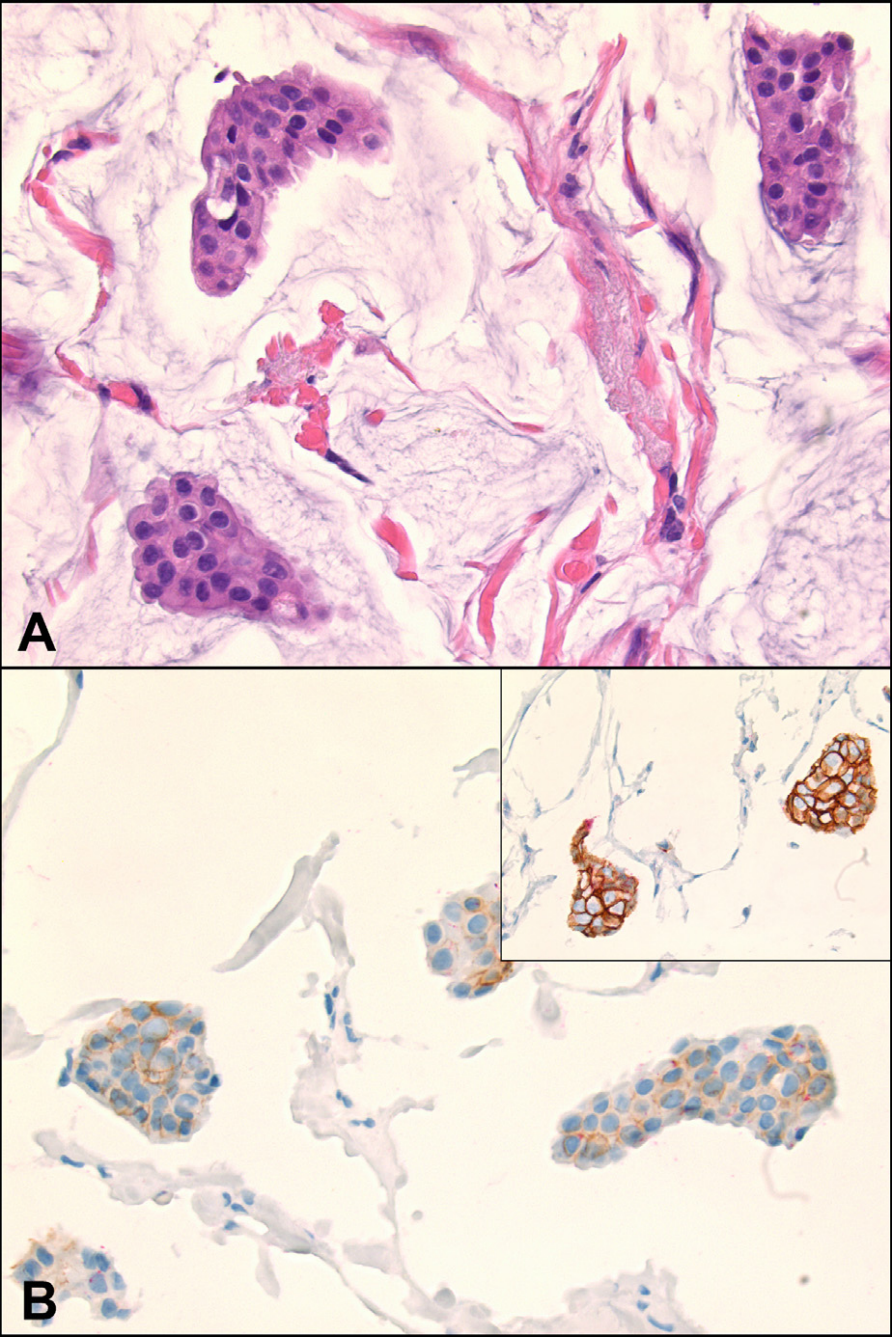
**Cases of pure mucinous carcinoma with significantly reduced membranous E-cadherin and p120 staining without redistribution of p120 into cytoplasm **(A, H&E, ×400; B, inset is a typical mucinous carcinoma with strong membranous E-cadherin staining, immunohistochemistry, ×400).

All 40 cases of carcinoma with mixed mucinous and non-mucinous components showed distinct membranous stainings for both E-cadherin and p120, depicting a clear ductal phenotype. However, 4 of the 40 cases (10%) of pure mucinous carcinoma demonstrated significant reduction in the membranous staining of E-cadherin without redistribution of cytoplasmic p120 staining (Figure 3). All 4 cases that showed reduced E-cadherin staining were nuclear grade 1 and negative for HER2 immunohistochemical stain.

## Discussion

Since the term *lobular carcinoma *was coined by Foote and Stewart in 1941 [14], it had long been accepted that the histologic pattern of lobular carcinoma was sufficiently distinct that a diagnosis could be comfortably made on the histologic sections. In the past three decades, the problems in reproducible diagnosis of invasive lobular carcinoma had been appreciated [15-17]. While the diversity of growth patterns is a major contributing factor to the reproducibility problem, the cytologic features of classical lobular carcinoma cells seem to have some value in recognizing the lobular phenotype. In our current case, the tumor growth pattern and cytomorphologic features are characteristic of a classical type ILC, even though the extracellular mucin secretion in ILCs has almost never been documented in the literature until a recent case report [18].

In most equivocal cases, immunohistochemical stains can help to solve the problem. Moll et al. demonstrated the loss of E-cadherin expression, an epithelial specific intracellular adhesion molecule, in lobular carcinoma [19]. The study by Dabbs et al. further indicated that the E-cadherin catenin complex was regularly disrupted in lobular neoplasia, manifested by the absence of E-cadherin in the cell membrane and redistribution of p120 catenin in the cytoplasm [20]. The absence of membranous E-cadherin staining and the diffuse cytoplasmic or punctuate paranuclear p120 staining are diagnostic of lobular phenotype [20-22]. The complete loss of membranous E-cadherin and the presence of diffuse cytoplasmic p120 staining pattern in our case unequivocally confirm the lobular phenotype of the tumor.

Mucin has been classified as membrane-bound mucin, which mediate signal transduction, and secretary mucin, which are directly secreted into extracellular spaces [23]. Mucinous breast lesions consist of a wide spectrum from benign fibrocystic changes to mucinous papillary lesions and mucinous carcinomas [4,24]. Extracellular mucin secretion is widely accepted as an indication of ductal phenotype, including solid papillary neoplasm, ductal carcinoma and mucinous carcinoma. In contrast, lobular carcinoma has been considered a variant of mucin-secreting carcinoma with only intracytoplasmic mucin [1-4].

Our case suggests that the lobular phenotype of breast carcinoma and the extracellular mucin secretion are not mutually exclusive. In spite of the presence of extracellular mucin, when the characteristic discohesive growth pattern and uniform cytology are present, a lobular carcinoma should be considered, and E-cadherin immunohistochemical stain should be performed to confirm the phenotype.

Intriguingly, none of the 80 TMAs of breast carcinoma with mucinous differentiation demonstrated clear immunohistochemical evidence of lobular phenotype, as it did in the current case. However, among the 80 cases, 4 of the pure mucinous carcinomas revealed significantly reduced membranous staining of both E-cadherin and p120, without redistribution of p120 into cytoplasm. Even though the significance of the finding is not clear at present, it confirms that the clear-cut lobular phenotype is a rare phenomenon in breast carcinomas with extracellular mucin production.

In the MUC gene family, the membrane-bound mucins include MUC1, MUC3, MUC4, among others; the secretary mucins include MUC2, MUC5AC, MUC6, among others [23]. Mucinous carcinoma of breast predominantly expresses the secretary mucins, MUC2 and MUC6 [4,24,25]. None of the ILCs and only a minority of invasive ductal carcinomas (IDCs) in the previous studies revealed MUC2 expression [24,25]. In contrast, overexpression of MUC1, which is present on the apical surface of normal secretary epithelium, has been demonstrated in the surface membrane of mucinous carcinoma cells, as well as in the cytoplasm of IDCs and intracytoplasmic vacuoles of ILCs [24,25].

The tumor in our case was negative for MUC2, MUC4, MUC5AC and MUC6. However, essentially all the tumor cells demonstrated cytoplasmic expression of MUC1, with some of the intracytoplasmic vacuoles being positive. This MUC1 staining pattern is similar to what has been observed in IDCs in some of the previous studies, in contrast to the predominant pattern of intracytoplasmic vacuoles in ILCs [24,25]; but it is similar to the study by Rahn et al., in which approximately half of the ILCs also showed strong cytoplasmic staining [26]. Numerous molecular and biochemical studies have demonstrated that MUC1 is involved in the inhibition of E-cadherin mediated cell-cell and cell-matrix adhesion [23,26-30]. The cytoplasmic domain of MUC1 molecule has been shown to inhibit the formation of E-cadherin - β-catenin complex [27,29,30]. Therefore, MUC1 may play a role in tumor invasion and metastases by disrupting cell adhesions. Disruption of E-cadherin-catenin complex is characteristic for lobular carcinomas, manifested as loss of E-cadherin immunoreactivity. As a consequence, invasive lobular carcinomas have a propensity for dissociated growth pattern and metastasis. Hence, it is not surprising to see overexpression of MUC1 in ILCs. However, the association between aberrant MUC1 expression pattern and phenotypic differentiation of breast carcinomas is still unclear.

In addition to the extracellular mucin production, another unusual presentation in our case is the amplification of *HER2 *gene in a classical ILC. The majority of ILCs, especially classical type, does not overexpress HER2 protein and belongs to luminal A or B molecular class [5-12]. In the only other case of classical ILC with extracellular mucin secretion in the literature, the tumor cells were negative for HER2 [18]. The four cases with reduced E-cadherin staining in our concurrent TMA study also showed negative HER2 expression. Our current case has drawn attention to the overlapping morphologic features as well as molecular manifestations between IDC and ILC. The morphological heterogeneity of breast carcinomas may, in fact, reflect the complex molecular pathogenesis of breast tumors.

Recent molecular and IHC studies have suggested that biomarker profile of an invasive cancer is likely more relevant for treatment purposes than subtyping tumors as ductal versus lobular. [7-10]. However, correct classification is important for uniform diagnosis, recognizing tumor recurrences, and understanding the biologic basis of the disease process.

Early genomic studies revealed very little overall difference in genomic profiles between low-grade IDC and classic ILC, implying that classic ILC might represent a subtype of low-grade IDC [31-34]. Recent gene expression studies comparing ILCs and IDCs have identified two subsets of ILCs with distinct transcription patterns [5,12]. Approximately half of the ILCs differ from IDCs in gene expression profiles ("typical" ILCs), while the remaining ILCs closely resemble IDCs in transcription patterns ("ductal-like" ILCs). On the other hand, a recent study on grade- and molecular subtype-matched ILCs and IDCs of no special type demonstrated that ILCs had different transcriptomic profiles in the genes related to cell-to-cell adhesion and signaling, as well as actin cytoskeleton signaling, when compared to grade- and molecular subtype-matched IDCs. This finding suggested that even though ILCs and IDCs might present as a spectrum or form a family, there were important differences that warrant classifying them into distinct entities [11].

In summary, we presented a case of classical type invasive lobular carcinoma with abundant extracellular mucin production, cytoplasmic MUC1 expression and unequivocal *HER2 *gene amplification. It brings awareness to the facts that extracellular mucin secretion is not an exclusive feature of ductal phenotype, and the classical morphologic definition of ductal and lobular carcinomas could sometimes be misleading. The differential expressions of E-cadherin and p120 in ductal and lobular carcinomas are useful tools to make the distinction in cases with mixed or unusual morphologic features. The phenotypic heterogeneity, including morphologic and immunohistologic variables, exists in lobular and ductal carcinomas, which may in fact reflect the molecular and genetic alterations in a comprehensive evolutionary pathway. More case observations and further studies are needed to document the biological behavior as well as molecular profiles associated with the phenotypic hybrids and their implications in clinical management.

## Competing interests

The authors declare that they have no competing interests.

## Authors' contributions

All authors have contributed to the content and design of this study. All authors read and approved the final manuscript.

## Consent

Written informed consent was obtained from the patient for publication of this case report and accompanying images. A copy of the written consent is available for review by the Editor-in-Chief of this journal.
